# Hepatic Alpha-1 Antitrypsin Globules in Compound Heterozygous SERPINA1 Variants Previously Considered Non-Polymerizing: A Case Report

**DOI:** 10.3390/ijms27125589

**Published:** 2026-06-20

**Authors:** Panaiotis Finamore, Simona Santangelo, Paolo Gallo, Ilaria Ferrarotti, Alice Maria Balderacchi, Andrea Falcomatà, Daniele Colombo, Franca Del Nonno, Umberto Vespasiani-Gentilucci, Raffaele Antonelli Incalzi, Simone Scarlata

**Affiliations:** 1 Unit of Internal Medicine, Fondazione Policlinico Universitario Campus Bio-Medico, 00128 Rome, Italyr.antonelli@policlinicocampus.it (R.A.I.);; 2 Operative Research Unit of Clinical Medicine and Hepatology, Fondazione Policlinico Universitario Campus Bio-Medico, 00128 Rome, Italy; 3 Laboratory of Molecular Pulmonology, UOC Pulmonology, IRCCS S. Matteo Hospital Foundation Pavia, 27100 Pavia, Italy; 4 Department of Internal Medicine and Therapeutics, Pulmonology Section, University of Pavia, 27100 Pavia, Italy; 5 Pathology Unit, National Institute for Infectious Diseases “Lazzaro Spallanzani” (IRCCS), 00149 Rome, Italyfranca.delnonno@inmi.it (F.D.N.)

**Keywords:** alpha-1 antitrypsin deficiency, gene expression, liver–lung interactions, M_procida_

## Abstract

Alpha-1 antitrypsin deficiency (AATD) is a genetically heterogeneous disorder with well-established pulmonary and hepatic manifestations; however, the clinical significance of rare compound heterozygous SERPINA1 variants remains incompletely defined. We report the case of a 61-year-old never-smoking woman with chronically elevated liver transaminase who was found to carry a compound heterozygous SERPINA1 genotype (PI*V/M_procida_) previously classified as non-polymerogenic and not previously associated with hepatic inclusions. This case expands the phenotypic spectrum of AATD and highlights the importance of considering SERPINA1 genotyping in adults with unexplained chronic transaminase elevation, while raising clinically relevant questions regarding surveillance and management in atypical AATD phenotypes.

## 1. Introduction

Alpha-1 antitrypsin (A1AT) deficiency is a hereditary genetic disorder characterized by reduced levels of the serine protease inhibitor A1AT and increased risk of respiratory and non-respiratory diseases. It is inherited in a codominant manner, meaning that both alleles of *SERPINA1* gene contribute to the final A1AT phenotype. Compound heterozygosity for SERPINA1 variants is uncommon, and its clinical implications for liver and lung disease are poorly defined. We report the case of a 61-year-old never-smoking woman with chronically elevated liver transaminase, liver involvement and apparent radiological progression of lung disease carrying a *SERPINA1* genotype previously classified as non-polymerogenic.

## 2. Detailed Case Description

**Figure 1:**
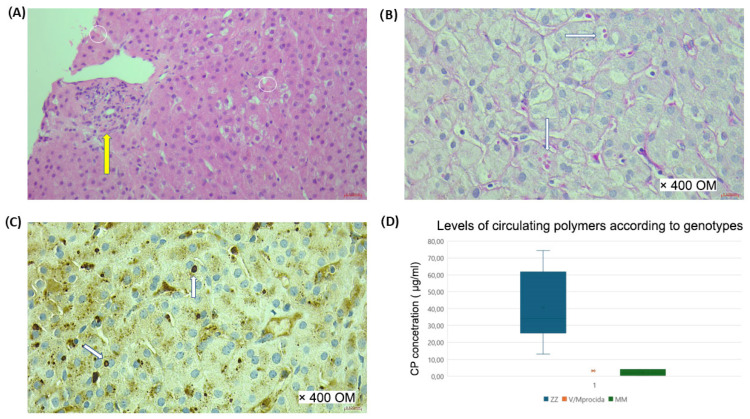
Panel (**A**): Liver parenchyma showing nonspecific features: mild inflammation of the portal tract (yellow arrow) with preserved bile duct. White circles highlight eosinophilic intracytoplasmic globules compatible with A1AT accumulation (hematoxylin-eosin stain, ×400 OM). Panel (**B**): A1AT globules are highlighted by the histochemical stain named periodic acid-Schiff with diastase (PAS-D), because the misfolded A1AT is diastase resistant and turns magenta (arrows) (×400 OM). Panel (**C**): Immunohistochemistry: Accumulation of A1AT protein as cytoplasmic globules (arrows) in liver cells with anti-alfa1-AT antibody (×400 OM). Panel (**D**): Serum concentration of circulating polymers (CP) in the patient with compound heterozygosity versus Pi*ZZ and Pi*MM patients.

The patient’s medical history included dyslipidaemia and hypertension, managed with ramipril and lercanidipine. There was no history of significant medical conditions during the neonatal period or childhood. She started in March 2019 a hepatological work-up for chronically elevated transaminase (1.5× the upper limit of normal). The patient reported no symptoms. On physical examination, the liver and spleen were not enlarged. HBV/HCV serology was negative, and there was no alcohol intake or hepatotoxic exposure. Autoantibodies revealed isolated ANA positivity (1:320, homogeneous pattern), with absent anti-mitochondrial (AMA), anti-liver–kidney microsomal type 1 (LKM1) and smooth muscle (SMA) antibodies. According to the rheumatology assessment, this finding did not warrant further investigation in the absence of symptoms, signs and other clinical features of autoimmune disease. Complete blood count, serum immunoglobulins, total IgE, gamma-globulins, ferritin and ceruloplasmin were normal. Abdominal ultrasonography showed no signs of chronic liver disease, steatosis or vascular abnormalities. The portal vein diameter was 5 mm, with hepatopetal flow and normal flow velocity (mean peak velocity 31 cm/s); hepatic veins were normal in caliber and anatomical course. Vibration-controlled transient elastography (VCTE) measured 6.2 kPa. Echocardiography showed normal biventricular function (ejection fraction 63%) without valvular disease. Given this picture, a liver biopsy was performed in October 2019, which showed nonspecific features of circulatory disorder, likely affecting venous outflow, and minimal cholangiopathy. The histochemical stain periodic acid-Schiff with diastase (PAS-D) and immunohistochemistry, performed with polyclonal anti-A1AT antibody, highlighted positive cytoplasmic globules (Figure 1A–C). These findings suggested a possible A1AT deficiency, prompting genetic testing that was carried out at IRCCS Policlinico San Matteo Pavia. Serum A1AT level was 89 mg/dL, with normal C-reactive protein, and the analysis confirmed compound heterozygosity in *SERPINA1* gene: V (p.G172R—c.514G>A—rs112030253) and M_procida_ (p.L65P—c.194C>T—rs28931569).

In May 2021, she was first referred to the Respiratory Outpatient Clinic of Fondazione Policlinico Universitario Campus Bio-Medico. Baseline chest CT scan (performed prior to presentation at our center) showed mild centrilobular and moderately bullous emphysema. Pulmonary function testing, performed according to ATS/ERS criteria in 2021, was within normal limits [FEV1/FVC 0.85 (107%), FEV1 2.37 L (91%), FVC 2.77 L (84%), TLC 4.39 L (85%), RV/TLC 42.22 L (114%), DLCO 11.71 mL/(min*mmHg) (59%), KCO 57%]. Given the patient’s genotype, absence of respiratory symptoms, and normal pulmonary function, augmented therapy was not indicated, and the patient entered semi-annual hepatology and annual respiratory follow-up. Ursodeoxycholic acid was started empirically.

At the respiratory follow-up in December 2024, spirometry and DLCO values remained stable, still within normal limits according to GLI reference values, with a FEV1 and FVC reduction of 60 and 80 mL, respectively. However, chest CT revealed two bullous lesions (largest measuring 12 mm) and some cylindrical bronchiectasis in the lower lobes. At the hepatology follow-up in July 2025, liver enzymes slightly improved and non-invasive assessment showed stable disease (VCTE 5.9 kPa; CAP 193 dB; IQR/median 13%). To further phenotype hepatic involvement, analysis of circulating A1AT polymers with a polymer-specific 2C1 monoclonal antibody (2C1mAb) was conducted [1], with no detectable polymers observed (Figure 1D). Currently, the patient continues with scheduled surveillance.

## 3. Discussion

No previous reports have linked liver involvement to these compound heterozygous SERPINA1 variants (PI*V/M_procida_). The M_procida_ mutation (p.L65P), first described in 1988 [2], is a deficient variant in exon 2 of the *SERPINA1* gene, whereas the V mutation (p.G172R), described in 1994 [3], is considered a benign (non-deficient) variant. Although M_procida_ has been associated with pulmonary emphysema, no in vivo study has previously reported an association between either the M_procida_ or the V variant and liver disease, leading to the classification of both as non-polymerogenic A1AT variants [4]. However, recent in vitro evidence suggests that the M_procida_ variant can form intracellular hepatic polymers, and that greater polymer accumulation is associated with a more severe secretory deficit [5]. The absence of detectable circulating polymers in this patient is consistent with recent findings by Balderacchi et al., who reported that heterozygous individuals carrying M/M_procida_ and M/V genotypes exhibited circulating polymer levels (normalized for A1AT concentration) comparable to those of M/M individuals. However, this observation contrasts with the patient’s evidence of liver disease with intrahepatic PAS-D+/A1AT+ globular inclusions. While PAS-D+ globules are not pathognomonic, as they have also been described in vascular conditions (e.g., Budd–Chiari syndrome, smoldering multiple myeloma or right-sided heart failure) [6], the absence of clinical, laboratory and imaging evidence for these entities, together with reduced serum A1AT levels, A1AT-positive liver immunochemistry and the identification of *SERPINA1* mutations supports A1AT deficiency as the most plausible cause of the hepatic inclusions in this patient. This apparent controversy relies in the behavior of M_procida_ variant. The substitution of the leucine with proline determines a mutation in the helices located on the opposite site to the central β-sheet A involved in Z mutations. Mutations occurring in that region, like M_wurzburg_, M_herleen_, and M_plowell_, regardless of the position of the amino acid substitution within the polypeptide chain, are not reactive to the 2C1mAb [5], used by Balderacchi et al., since it recognizes a cryptic epitope that is exposed by polymers formed by the canonical Z AAT polymerization pathway. Therefore, it can be concluded that the apparent absence of circulating polymers may simply reflect the inability of the 2C1mAb antibody to detect them. The absence of polymer-specific staining and conformationally independent quantification of the intrahepatic aggregates prevents us from determining the exact nature of the patient’s A1AT-positive globular inclusions. These inclusions may be composed of homopolymers of the deficient M_procida_ variant; however, the presence of M_procida_/V heteropolymers, although less likely given the benign nature of the V variant, cannot be completely ruled out. Indeed, some rare missense variants, although not overtly polymerogenic in standard assays, may subtly impair folding kinetics, conformational flexibility or stability sufficiently to cause slow intracellular aggregation/retention under the stress of compound heterozygosity. A well-known precedent is the S variant, which is recognized for its increased susceptibility to polymerization, though it is efficiently degraded and does not accumulate in the liver, even in homozygous individuals (PI*SS) [7,8]. However, when inherited in compound heterozygosity with the Z variant, it contributes to an increased risk of liver disease [9] due to the formation of heteropolymers [8].

The present case is the first describing a hepatic A1AT-positive globules in a patient carrying SERPINA1 variants usually classified as non-polymerogenic. From a clinical standpoint, this is consistent with recent hepatology guidance on genetic cholestatic liver diseases. EASL guideline lists A1AT deficiency among inherited cholestatic disorders that can present in adulthood and underscores appropriate diagnostic testing in suspected atypical cases (serum A1AT and phenotyping/genotyping) [10]. Management of A1AT deficiency-related liver disease remains supportive and surveillance-based. There is insufficient evidence to advise for/against UDCA [10], and there is currently no approved treatment for liver manifestations. Augmentation therapy is ineffective in A1AT-related liver disease, and novel therapeutic approaches aimed at correcting or stabilizing the folding of deficient variants are warranted.

## 4. Conclusions

We describe a case of chronically elevated liver transaminase with histologic findings of A1AT inclusions in an adult with compound heterozygous SERPINA1 variants (PI*V/M_procida_) previously classified as non-polymerogenic. Furthermore, it is worth noting that this patient, despite having normal spirometry, demonstrated the development of bronchiectasis and two bullous lesions in the absence of smoking or other recognized environmental risk factors. If the apparent radiological progression was confirmed by more accurate examinations, such as CT densitometry, it would raise the question of whether treatment should be considered in individuals who exhibit radiologic progression despite preserved pulmonary function, a scenario that is not currently addressed by the ERS statement [11].

## Data Availability

All relevant data are included within the article. Additional data are not publicly available in order to protect patient confidentiality.
